# *In vitro* antibacterial activity and *in vivo* therapeutic effect of *Sesbania grandiflora* in bacterial infected silkworms

**DOI:** 10.1080/13880209.2017.1297467

**Published:** 2017-03-02

**Authors:** Pimporn Anantaworasakul, Hiroshi Hamamoto, Kazuhisa Sekimizu, Siriporn Okonogi

**Affiliations:** a Department of Pharmaceutical Sciences, Faculty of Pharmacy, Chiang Mai University, Chiang Mai, Thailand;; b Institute of Medical Mycobiology, Teikyo University, Tokyo, Japan

**Keywords:** Antibacterial extract, drug-resistant organisms, silkworm infection, killing rate

## Abstract

**Context:** Antibiotic resistance is a serious problem worldwide. Searching for new potential agents is, therefore, essential. The bark of *Sesbania grandiflora* (L.) Pers. (Fabaceae) has been used in folk medicine against various diseases.

**Objective:** To investigate the antibacterial activity of *S. grandiflora* bark and explore the therapeutic effect of the highest potent fraction.

**Materials and methods:** Bacteria and healthy silkworms were exposed to three fractionated extracts (3.1–400 mg/mL) of *S. grandiflora* bark from hexane (HXF), chloroform (CFF), and ethyl acetate (EAF). The sets of bacteria were incubated at 37 °C while silkworms were kept at 27 °C for 24 h. To evaluate the therapeutic effect, silkworms infected with bacteria were exposed to the extracts (0.5–60 mg/mL) and incubated at 27 °C for 52 h. Qualitative analysis of the most potent extract was done using HPLC.

**Results:** EAF showed the highest activity with MIC against methicillin resistant *Staphylococcus aureus* (MRSA) and vancomycin resistant enterococci (VRE) of 1.6 and 0.4 mg/mL, respectively, and against Gram-negative *Escherichia coli* and *Pseudomonas aeruginosa* of 6.2 and 3.1 mg/mL, respectively. It is nontoxic to silkworms with LC_50_ >400 mg/mL and has high therapeutic effect on infected silkworms with EC_50_ of 1.9 mg/mL. EAF consists of at least five major compounds, one of them is gallic acid. The activity of EAF is higher than the sum of individual activities of separated compounds.

**Discussion and conclusion:** These results suggest that EAF is a promising antibacterial extract, suitable for further investigation in rodents infected with drug resistant bacteria.

## Introduction

Antibiotic resistance is currently a serious and widespread problem in both developing and developed countries causing high mortality every year (Cosgrove & Carmeli 2003; Gyles 2011). Despite the use of advanced antibiotics, the infectious diseases remain an important cause of morbidity and mortality. Moreover, the synthetic drugs may expose the patients to the risk of harmful and side effects. Medicinal plants have been used for centuries as remedies for human diseases. They contain components, usually are their secondary metabolites, which have various pharmacological properties (Tachakittirungrod et al. 2007; Saeio et al. 2011; Chaiyana et al. 2013; Okonogi et al. 2013). Some of them have been used as the active ingredients e.g. morphine from *Papaver somniferum* L. (Papaveraceae) for pain treatment (Pace & Burke 1996; Sverrisdóttir et al. 2015) and colchicine from *Colchicum autumnale* L. (Colchicaceae) for treatment of pericardial disease (Deftereos et al. 2013). Therefore, searching for therapeutic agents from plants is an alternative way to solve these problems. Indeed, certain plant extracts show significant potential against drug resistant bacteria (Padhi & Panda 2015; Valle et al. 2015; Dzotam et al. 2016).

*Sesbania grandiflora* (L.) Pers. (Fabaceae) is commonly found in Thailand and other Asian countries. The aerial part of this plant are edible and have been used as folk medicine in the treatment of various diseases related to bacterial infection such as skin disorders, ulcers, and wounds (Sertie et al. 2001; Karthikeyan et al. 2011; Venkateshwarlu et al. 2012). Leaves and flowers of this plant were reported to have anticancer (Sreelatha et al. 2011), anxiolytic (Kasture et al. 2002), and antioxidant activities (Shyamalagowri & Vasantha 2010). Its bark extract was reported to have high inhibitory activity on acute inflammation and adjuvant-induced arthritis in rats (Patil et al. 2010; Ouattara et al. 2011). In our project of searching for antibacterial phytomedicine from plants used in Thai folk medicine, we therefore targeted this plant. We previously reported the comparative antibacterial activity of the extracts from different parts of this plant and found that the extract from its bark was the most effective (Anantaworasakul et al. 2011). In the present study, we used only the bark of this plant but extracted fractionally with different solvents and compared the activity among the obtained extracts against clinical pathogenic bacteria.

Moreover, the extracts were also *in vitro* tested with many strains of drug resistant bacteria. Silkworm is one of invertebrate animals that have been used as an animal model. The silkworm has several advantages as a model for the study of therapeutic effects of antibiotics. The size of silkworms is large enough to handle injection of fixed volumes of sample solution with syringes into the hemolymph of the silkworm (Asami et al. 2010). Quantitative evaluation of the therapeutic effect of antibiotics in silkworm is consistent with dose in mammalian systems (Hamamoto et al. 2004; Fujiyuki et al. 2010). Furthermore, toxicity of compounds can be evaluated using the silkworm model (Hamamoto et al. 2009). Previous studies reported that silkworms infected by pathogenic bacteria could be treated with antibiotics (Kaito et al. 2002). We recently identified a novel antibiotic, Lysocin E, from a culture supernatant of *Lysobacter* bacteria, by monitoring therapeutic activity in the silkworm infection model with *Staphylococcus aureus* (Hamamoto et al. 2015).

The present study explores the antibacterial activity of the bark extracts of *S. grandiflora* against more potent pathogenic bacteria, particularly drug resistant strains. Therapeutic effect and toxicity of the extracts were investigated in bacterial infected silkworms. In addition, a marker active compound of the most potential extract was also identified.

## Materials and methods

### Chemicals and reagents

Gallic acid, sodium chloride, and dimethyl sulfoxide (DMSO) were purchased from Sigma-Aldrich (St. Louis, MO). Vancomycin was from Shionogi & Co., Ltd. (Osaka, Japan). Gentamicin was from Sankyo Co., Ltd. (Tokyo, Japan). Organic solvents were from Merck (Darmstadt, Germany) and Wako Pure Chemicals (Osaka, Japan). Cation-adjusted Muller-Hinton broth (MHB) was from DIFCO (Franklin Lakes, NJ). All other chemicals were of the highest quality grade available.

### Plant material

Bark sample of *S. grandiflora* was collected in February 2014 from Chiang Mai province, a northern area of Thailand. The plant species was identified by the botanist of the Biomedical Engineering Center, Chiang Mai University, Chiang Mai, Thailand. A voucher specimen (no. 023207) was deposited at the Herbarium of the Faculty of Pharmacy, Chiang Mai University, Chiang Mai, Thailand. The bark was dried at 50 °C for 48 h and ground into powder.

### Animals

Silkworms (*Bombyx mori* L.) of Japanese breeding at the state of the fifth-instar larvae having body weight of 1.7 ± 0.1 g were used. Preparation of silkworms prior to the tests was done as in a previous report with some modification (Anantaworasakul et al. 2013). Briefly, the silkworms were reared on an antibiotic-free artificial diet and kept overnight at 27 °C until further experiments. Two-day-old fifth-instar larvae were used for evaluation of the toxicity and therapeutic effect of the extracts.

### Preparation of plant extracts

Three different extracts of *S. grandiflora* bark were prepared according to the method previously described (Okonogi et al. 2013). Briefly, the dried powder of *S. grandiflora* bark was macerated for 24 h in hexane, then filtered through a filter paper. The solid residue after filtration was dried in open air and further macerated with chloroform, and then ethyl acetate, respectively, in the same manner as hexane. The solvent from different filtrate was removed under vacuum using a rotary evaporator at 40 °C. The dried fractionated bark extracts of hexane (HXF), chloroform (CFF), and ethyl acetate (EAF) were kept in tight containers in the refrigerator for further studies.

### *In vitro* antibacterial study

The *in vitro* antibacterial activity of the extracts was investigated by broth micro-dilution assays in order to determine the minimum inhibitory concentration (MIC). The bacteria used in this study were clinical pathogenic sensitive and resistant strains of Gram-positive and Gram-negative bacteria as shown in Table 1. Strains of *S. aureus* were isolated in Kyushu University Hospital, Fukuoka, Japan. *Streptococcus* species were isolated at the University of Tokyo Hospital, Tokyo, Japan. *Listeria monocytogenes* and *Enterococcus faecalis* were received from the National Institute of Infectious Diseases, Tokyo, Japan. All other strains were obtained from our laboratory stocks. The extracts were dissolved in MHB containing 10% DMSO to a concentration of 400 mg/mL for aerobic or facultative organisms, and that containing 2.5% lysed horse blood for fastidious organisms. The stock solutions of the tested extracts were centrifuged at 14,000 rpm for 1 min to remove some undissolved particles. The supernatant was diluted with MHB into 2-fold serial dilutions and added with an equal volume of the bacterial suspension having a concentration equal to 100-fold dilution of McFarland No. 0.5, and then incubated at 37 °C for 24 h. The final concentrations of the extracts exposed to bacteria were 0.1, 0.2, 0.4, 0.8, 1.6, 3.1, 6.2, 12.5, 25, 50, 100, and 200 mg/mL. The minimum concentration of the samples which could inhibit the bacterial growth was recorded as MIC. Vancomycin and gentamicin were used as standard antibacterial controls. The final concentrations of these drugs were 0.06, 0.1, 0.3, 0.5, 1, 2, 4, 8, 16, 32, 64, and 128 μg/mL.

**Table 1.** Bacteria used in *in vitro* antibacterial study.

Bacterial strains	Gram stain	Drug sensitivity	Type
*Staphylococcus aureus* (MSSA1)	Positive	Sensitive	Facultative
*S. aureus* (MRSA3)	Positive	Resistant (methicillin)	Facultative
*S. aureus* (MRSA4)	Positive	Resistant (methicillin)	Facultative
*S. aureus* (MRSA6)	Positive	Resistant (methicillin)	Facultative
*S. aureus* (MRSA8)	Positive	Resistant (methicillin)	Facultative
*S. aureus* (MRSA9)	Positive	Resistant (methicillin)	Facultative
*S. aureus* (MRSA11)	Positive	Resistant (methicillin)	Facultative
*S. aureus* (MRSA12)	Positive	Resistant (methicillin)	Facultative
*Bacillus subtilis* JCM2499	Positive	Sensitive	Facultative
*B. cereus* JCM 20037	Positive	Sensitive	Aerobic
*Streptococcus sanguinis*	Positive	Sensitive	Facultative^a^
*Strep. pneumoniae*	Positive	Sensitive	Facultative^a^
*Strep. agalactiae*	Positive	Sensitive	Facultative^a^
*Strep. pyogenes*	Positive	Sensitive	Aerobic^a^
*Listeria monocytogenes*	Positive	Sensitive	Facultative^a^
*Enterococcus faecalis* EF1	Positive	Sensitive	Facultative^a^
*E. faecalis* EF5 (VRE)	Positive	Resistant (vancomycin)	Facultative^a^
*Escherichia coli* w3110	Negative	Sensitive	Facultative
*Pseudomonas aeruginosa* PA01	Negative	Sensitive	Facultative

a Fastidious bacteria.

### Toxicity test

This test was performed and evaluated according to the previous report (Liu et al. 2014) with some modification. Briefly, the extract was dissolved in sterile normal saline solution (NSS) containing 10% DMSO (DMSO-NSS) to have a concentration of 400 mg/mL. The obtained dispersion was centrifuged at 14,000 rpm for 1 min, to remove undissolved particles. The supernatant was diluted with NSS to obtain series of 3.1, 6.2, 12.5, 25, 50, 100, 200, and 400 mg/mL. Silkworms were divided into five batches, two batches for two controls (vehicle and no treatment), and three batches for the three different test extracts (HXF, CFF, and EAF). Each batch of the test extracts was divided into eight groups according to the eight different extract concentrations, each group consisting of 10 silkworms. The exact amount of 0.05 mL of each extract dilution was injected into the hemolymph through the dorsal surface of the silkworms using a 27-gauge needle. The vehicle control group was injected with 0.05 mL of 10% DMSO in NSS, and the no treatment control group was not injected. After that, all silkworms were kept at 27 °C. The survival of the silkworms was observed within 24 h.

### Therapeutic effect

Strain of *S. aureus* (MSSA1) sub-cultured in MHB was incubated overnight at 37 °C. The bacterial suspension was prepared by diluting the cells with NSS to a desired concentration of McFarland turbidity standard No. 0.5. Freshly prepared suspension was used throughout the test. Silkworms were divided into five batches, two batches for two controls (vehicle and antibiotic), and three batches for the three different test extracts (HXF, CFF, and EAF). Each batch of the test extracts was divided into eight groups according to the eight different extract concentrations, each group consisting of 10 silkworms. The exact volume of 0.05 mL of bacterial suspension was injected into the silkworm hemolymph and incubated at 27 °C for 1 h. The bacterial infected silkworms were then injected into the hemolymph with the tested extracts at concentrations of 0.5, 0.9, 1.9, 3.8, 7.5, 15, 30, and 60 mg/mL, prepared same procedure as in toxicity test (Kaito et al. 2002; Hamamoto et al. 2009). NSS was used as a vehicle control while 100 μg/mL vancomycin was used as antibiotic control. The silkworms were further incubated at 27 °C and observed for their survival during 52 h.

### Qualitative HPLC

The most potent extract was analyzed by qualitative HPLC analysis using a Hypersil ODS column (4.6 i.d. × 250 mm) and gradient eluent of solvent A (acetic acid-water, 1:99 v/v) and solvent B (methanol). The eluent gradient program was started with 100% of solvent A for 1 min, and then changed to 70% and 40% at 10 and 20 min, respectively. Finally, the eluent composition was brought back to 100% of solvent A in 5 min for the next run. The eluent was detected by an UV/VIS detector at wavelengths of 280 nm using a flow rate of 1 mL/min. The chromatogram of the extract was compared with that of a standard of gallic acid injected in the same HPLC conditions.

### Preparative HPLC

In this experiment, the most potent extract was dissolved in methanol and then subjected to preparative HPLC using a Senshu Pak PEGASIL ODS SP100 column (20 i.d. × 250 mm). The ambient temperature of the ODS column was maintained at 40 °C. The mobile phase was a linear gradient from 10% to 100% of methanol after 30 min, and was eluted with 100% methanol for 15 min at a flow rate of 9 mL/min. The eluent of each fraction was examined by a photodiode array detector (PDA) and collected every 2 min. Each eluent was evaluated for antimicrobial activity in comparison with the unseparated extract before injection.

### Statistical analysis

The obtained data of *in vitro* antibacterial activity and the silkworm toxicity tests were analyzed statistically using the Student's *t*-test as performed by SPSS statistic 17.0 software. For therapeutic effect evaluation, the Log-rank test as performed by Prism5 for Mac OS X (GraphPad Software, Inc.) was used. Differences at *p <* 0.05 were considered significant.

## Results

### *In vitro* antibacterial activity

All bacterial strains used in this study were human pathogens including drug-sensitive and drug-resistant bacteria. It was found that HXF, CFF, and EAF possessed inhibitory activity against all tested pathogenic microorganisms but at different levels (Table 2). Among the three extracts, EAF had the strongest activity. EAF showed the highest inhibition on the drug resistant strains such as VRE with the MIC of only 0.4 mg/mL and MRSA with the MIC of 1.6 mg/mL, approximately 15–125 times higher activity than HXF and CFF. Moreover, EAF showed high activity against Gram-negative pathogenic bacteria with the MIC of 3.1–6.2 mg/mL, approximately 16–65 times higher activity than HXF and CFF. Vancomycin exhibited antibacterial activity against most of the tested Gram-positive bacteria with MIC of less than 2 μg/mL except for VRE of which the MIC was 128 μg/mL, and the drug did not present good activity against Gram-negative bacteria in comparison with gentamicin. This drug displayed strong inhibitory activity against Gram-negative bacteria like *E. coli* w3110 and *P. aeruginosa* PA01 with the MIC of only 0.5 and 8 μg/mL but did not show good activity against Gram-positive bacteria, particularly most of the drug resistant strains like MRSA and VRE.

**Table 2.** MIC of the extracts and the standard drugs.

	MIC				
Bacterial strains	HXF (mg/mL)	CFF (mg/mL)	EAF (mg/mL)	Vancomycin (μg/mL)	Gentamicin (μg/mL)
MSSA1	50	100	1.6	0.5	1
MRSA3	25	25	1.6	1	128
MRSA4	50	50	1.6	1	128
MRSA6	25	25	1.6	1	128
MRSA8	50	50	1.6	1	128
MRSA9	100	25	1.6	1	128
MRSA11	50	25	1.6	1	128
MRSA12	50	50	1.6	1	0.1
*B. subtilis* JCM2499	50	50	0.8	0.3	0.1
*B. cereus* JCM 20037	25	25	0.8	0.3	0.5
*Strep. sanguinis*	3.1	1.6	1.6	1	4
*Strep. pneumoniae*	100	100	1.6	1	4
*Strep. agalactiae*	100	50	1.6	1	4
*Strep. pyogenes*	1.6	6.2	1.6	1	4
*L. monocytogenes*	100	50	3.1	2	0.3
*E. faecalis* EF1	25	100	1.6	1	16
VRE	25	50	0.4	128	128
*E. coli* w3110	100	200	6.2	>128	0.5
*P. aeruginosa* PA01	100	200	3.1	>128	8

### *In vivo* toxicity and therapeutic effect

**Figure 1.**
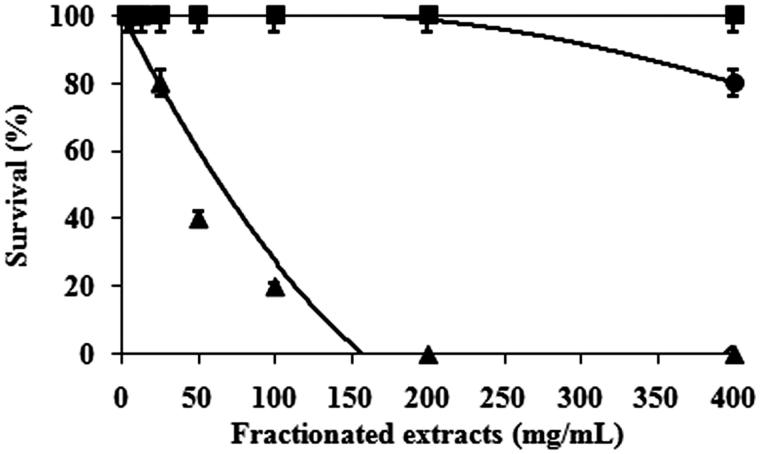
Survival of silkworms injected with the sequentially fractionated extracts of *S. grandiflora* bark from hexane (HXF) (▪), chloroform (CFF) (▴), and ethyl acetate (EAF) (•). After injection, all silkworms were kept at 27 °C for 24 h. Numbers of surviving silkworms were counted.

**Figure 2.**
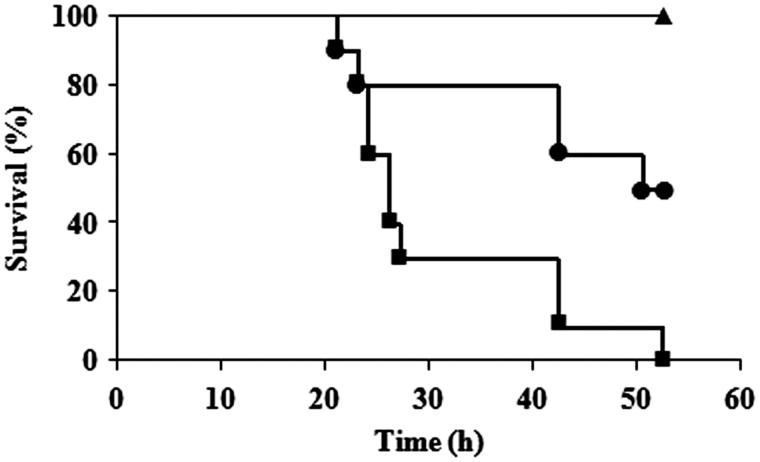
Survival curve of silkworms infected with *S. aureus* and injected with saline (▪), 95 μg of EAF (•), and 5 μg of vancomycin (▴). Significant difference was determined by log-rank test (*p <* 0.05).

In the toxicity study, the aqueous dispersion containing the tested extract of 3.1, 6.3, 12.5, 25, 50, 100, 200, 400 mg/mL was used in order to investigate the concentration at which 50% of silkworms were killed (LC_50_). The results revealed that LC_50_ of CFF was 65.9 mg/mL, significantly different from those of HXF and EAF which were higher than 400 mg/mL as shown in Figure 1. The numbers of dead silkworms injected with CFF was dose dependent. All silkworms receiving HXF survived even in the highest tested concentration of 400 mg/mL whereas 80% of the silkworms injected with EAF survived at this highest dose. Therefore, HXF and EAF were considered to be nontoxic where CFF was characterized as toxic to the silkworms. According to this toxicity results, the concentration of the three extracts used for therapeutic investigation was not exceed than the LC_50_ of CFF. All infected silkworms injected with NSS gradually died during 52 h (Figure 2). All infected silkworms injected with every tested concentration of CFF and HXF were killed at the same rate as those receiving NSS. EAF showed an obvious therapeutic effect on the infected silkworms and the effect was dose dependent. Figure 2 showed that 50% of the infected silkworms injected with 1.9 mg/mL EAF and 100% silkworms injected with vancomycin could survive after 52 h of injection. It was also found that 80% and 100% of the infected silkworms could survive after injection with EAF of 3.8 and 7.5–60 mg/mL, respectively. The 50% effective concentration (EC_50_) of EAF was considered to be 1.9 mg/mL.

### HPLC analysis

**Figure 3.**
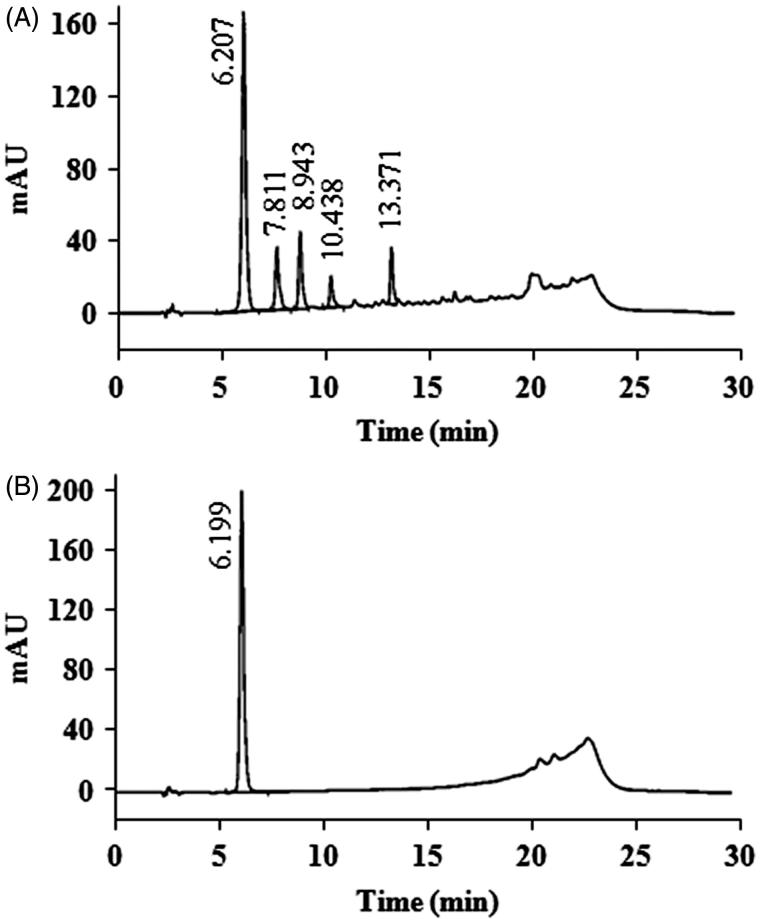
Qualitative HPLC chromatogram of EAF (A) and standard gallic acid (B), detected at 280 nm.

**Figure 4.**
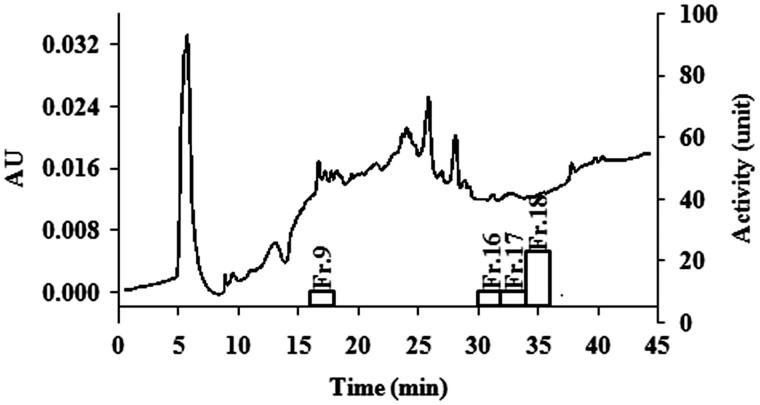
Preparative HPLC consequences of the EAF detected at 210 nm and antibacterial activity of each 2-min eluent fraction. The line represents the intensity of peaks (left axis) whereas the column represents antibacterial activity (right axis).

**Figure 5.**
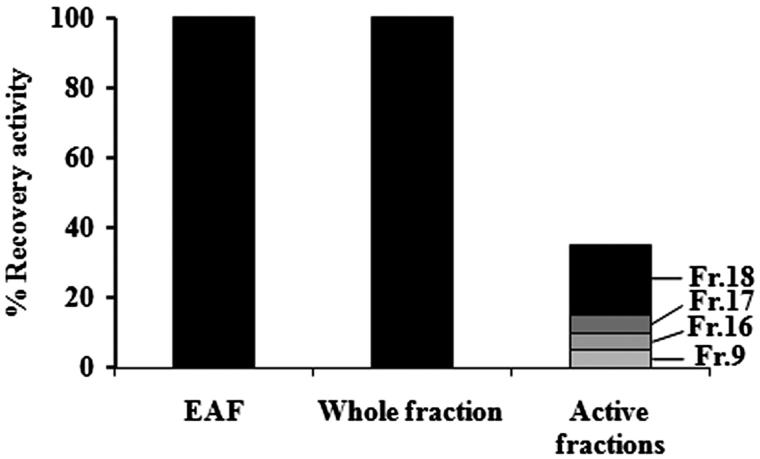
Antibacterial activity of EAF (unseparated extract), whole fraction (total of eluent fractions which were separated from EAF), and different active fractions from the preparative HPLC; fraction 9 (Fr.9), fraction 16 (Fr.16), fraction 17 (Fr.17), and fraction 18 (Fr.18) (getting individually each 2 min eluent fractions which separated from EAF).

EAF was selected for qualification because it presented the highest antibacterial activity. The HPLC fingerprint of EAF as shown in Figure 3(A) demonstrated a major peak at a retention time of 6.207 min. This peak was comparable with the HPLC chromatogram of gallic acid which eluted at significantly the same retention time of 6.199 min (Figure 3(B)) after running under the same conditions and eluting detection. EAF was separated by preparative HPLC for quantitative analysis. Various fractions were collected every 2 min and tested for antibacterial activity using *S. aureus* (MSSA1) as a test strain. The results demonstrated that EAF consisted of many weak antibacterial compounds as shown in Figure 4. Preparative HPLC consequences of EAF also indicated that each of fractions 9, 16, and 17 had approximately 5% of the total antibacterial activity of EAF whereas fraction 18 contributed 20% of the activity. However, the entirely collected eluent as sum of different active fractions showed 100% antibacterial activity as shown in Figure 5.

## Discussion

The searching for new antibacterial agents is currently important according to high incidence of antibiotic resistance among pathogenic bacteria such as MRSA and VRE which are resistant to methicillin and vancomycin, respectively (Boucher et al. 2009). Certain strains of *S. aureus* and enterococcus were reported to habituate to gentamicin, so these pathogens also showed a proportional increase in resistance to gentamicin (Dawson 2002; Boucher et al. 2009). The treatment of resistant bacterial infection nowadays is complicated. Many remedies use several antibiotics in combination with the prospect of synergistic antibacterial action (Mulazimoglu et al. 1996; Lee et al. 2003). However, the incidence of drug resistance has rapidly increased and causes a serious problem in clinical therapy. New generations of antibiotics which are capable of treating such antibiotic resistant bacterial strains are being manufactured (Kaito et al. 2002; Hamamoto et al. 2009). The research of alternative and effective phytochemicals against such pathogens from potential medicinal plants which are widely available, less toxic, and less expensive has become an important concern all over the world (Kenny et al. 2014; Rubens et al. 2015). In addition, misuse of antibiotics is one of the major causes of widespread drug resistance. The rationale for using antibacterial plant extracts was to decrease the problems of over prescription and misuse of antibiotic drugs (Moloney 2016). Medicinal plants in the Asian region including Thailand have been demonstrated to have high antibacterial activity against pathogenic strains (Okonogi et al. 2011; Prakatthagomol et al. 2011; Sakunpak & Panichayupakaranan 2012). Some of these show high ability to inhibit the growth of drug resistant strains such as *Punica granatum* L. (Lythraceae) (Voravuthikunchai & Kitpipit 2005), *Quercus infectoria* Oliv. (Fagaceae) (Voravuthikunchai & Mitchell 2008), and *Garcinia mangostana* L. (Clusiaceae) (Chomnawang et al. 2009).

In the current study the antibacterial activity of *S. grandiflora* bark extracts obtained from different fractions according to the three extracting solvents is explored. The *in vitro* antibacterial assay used in the present study was the microbroth dilution assay. This assay is particularly appropriate for *in vitro* antimicrobial activity evaluation of plant extracts because of its high reproducibility compared with the agar diffusion method (Wilkinson 2006). EAF also revealed high activity against the tested drug-resistant strains. The MIC of EAF against MRSA and VRE were 1.6 and 0.4 mg/mL, respectively. EAF show significantly higher activity than the plant previously reported [*Lantana camara* L. (Verbenaceae)] which its dichloromethane extract, which showed MIC against MRS and VRE of 6.25 mg/mL and its methanolic extract showed MIC against these two pathogens of 3.125 and 6.25 mg/mL, respectively (Dubey & Padhy 2013).

These results provide important scientific evidence to support the use of *S. grandiflora* bark as an active ingredient in Asian folk medicine remedies to treat ulcers and wounds as well as other skin disorders related to infections (Venkateshwarlu et al. 2012). However, results of antibacterial activity obtained from *in vitro* study are usually not sufficient to indicate the therapeutic effect in human due to issues of toxicity and pharmacodynamics. Consequently, pre-clinical testing in animal models is essential for evaluating toxicity and therapeutic effect of the active agents. There are several studies reported on the advantages of using silkworms for therapeutic evaluation in the first step of *in vivo* search for new antibacterial agents (Kaito et al. 2002; Hamamoto et al. 2004, 2009, 2015; Asami et al. 2010; Fujiyuki et al. 2010). The *in vivo* results demonstrate that EAF possesses highly effective therapeutic action against *S. aureus* in infected silkworms. This extract exhibited not only prolonged survival of the infected silkworm but also showed nontoxicity to the silkworms. Previous report revealed that the antibacterial property of *S. grandiflora* root was caused by several phytoconstituents in the extract (Manigandan & Muzammil 2013). The HPLC fingerprint of EAF was constructed in the current study and indicated that several constituents exist. One major peak was eluted at the same retention time as gallic acid under the same HPLC conditions. Gallic acid is a phenolic compound and possesses inhibitory activity against various microorganisms including bacteria (Díaz-Gómez et al. 2014; Cossu et al. 2016). It is therefore proposed that a major compound of EAF might be gallic acid. Besides, when fractions from the preparative HPLC were tested for antibacterial activity, it was found that the activity of each fraction was not high. The sum of recovery activity of those fractions was only 35% whereas the recovery of antimicrobial activity was 100% when the eluent of the chromatography was collected in total. Previous studies reported the activity enhancement as synergism from the combination of more than one active ingredient could occur (Zuo et al. 2011; Naksuriya & Okonogi 2015). It is considered that a synergistic effect of phytochemical compounds existing in EAF causes the strong antibacterial activity.

## Conclusions

Among three fractionated extracts of *S. grandiflora* bark, EAF possesses the highest antibacterial activity against drug sensitive and resistant pathogenic bacteria (*p <* 0.05). The MIC of EAF against drug resistant bacteria, MRSA and VRE, are 1.6 and 0.4 mg/mL, respectively. EAF also inhibits Gram-negative *E. coli* and *P. aeruginosa* with MIC of 6.2 and 3.1 mg/mL, respectively. EAF and HXF are nontoxic (LC_50_ >400 mg/mL) to the silkworms in comparison with CFF (LC_50_ = 65.9 mg/mL). HXF and CFF have no therapeutic effect whereas EAF has a significant (*p <* 0.05) therapeutic effect on infected silkworms with EC_50_ of 1.9 mg/mL. EAF consists of at least five compounds, one of them being gallic acid. Preparative HPLC indicates that a synergistic effect of several compounds in EAF results (*p <* 0.05) in antibacterial activity higher than the sum of individual activities. These results suggest that EAF is a promising antibacterial extract and suitable for further investigation in rodents infected with drug resistant bacteria.
